# Mast cells increase adult neural precursor proliferation and differentiation but this potential is not realized *in vivo* under physiological conditions

**DOI:** 10.1038/s41598-017-18184-2

**Published:** 2017-12-19

**Authors:** Joanna M. Wasielewska, Lisa Grönnert, Nicole Rund, Lukas Donix, Ruslan Rust, Alexander M. Sykes, Anja Hoppe, Axel Roers, Gerd Kempermann, Tara L. Walker

**Affiliations:** 1 CRTD – Center for Regenerative Therapies Dresden, Technische Universität Dresden, Dresden, Germany; 2 German Center for Neurodegenerative Diseases (DZNE) Dresden, Dresden, Germany; 3 Brain Research Institute ETH and University of Zurich, Zurich, Switzerland; 4 Max Planck Institute for Molecular Cell Biology and Genetics, Dresden, Germany; 5 Institute for Immunology, Medical Faculty Carl Gustav Carus, Technische Universität Dresden, Dresden, Germany

**Keywords:** Neurogenesis, Neuroimmunology

## Abstract

There is growing evidence that both peripheral and resident immune cells play an important part in regulating adult neural stem cell proliferation and neurogenesis, although the contribution of the various immune cell types is still unclear. Mast cells, a population of immune cells known for their role in the allergic response, have been implicated in the regulation of adult hippocampal neurogenesis. Mast cell-deficient c-kit^W-sh/W-sh^ mice have previously been shown to exhibit significantly decreased adult hippocampal neurogenesis and associated learning and memory deficits. However, given that numerous other cell types also express high levels of c-kit, the utility of these mice as a reliable model of mast cell-specific depletion is questionable. We show here, using a different model of mast cell deficiency (Mcpt5CreR26^DTA/DTA^), that precursor proliferation and adult neurogenesis are not influenced by mast cells *in vivo*. Interestingly, when applied at supraphysiological doses, mast cells can activate latent hippocampal precursor cells and increase subventricular zone precursor proliferation *in vitro*, an effect that can be blocked with specific histamine-receptor antagonists. Thus, we conclude that while both mast cells and their major chemical mediator histamine have the potential to affect neural precursor proliferation and neurogenesis, this is unlikely to occur under physiological conditions.

## Introduction

Peripheral immune cells and the factors they secrete can influence adult neurogenesis, not only in the context of disease but also under baseline conditions^1–5^. Mast cells (MC), widely known for their role in allergy and histaminergic responses^6,7^, are derived from hematopoietic stem cells and reside in small numbers within the brain from birth. During postnatal development, MC migrate along blood vessels of the hippocampus and fimbria and penetrate into the thalamus, where they remain throughout adulthood. This may suggest an affinity of MC to populate brain regions characterized by a high degree of adaptive rewiring and structural rearrangement during life^8^. Being part of both the innate and adaptive immune systems, MC serve as sensors and effectors in the crosstalk between nervous, vascular and immune systems. Within the brain, they interact with microglia, astrocytes, neurons and blood cells to initiate, amplify and prolong other immune and nervous responses. Their versatile pro-inflammatory, anti-inflammatory and immunosuppressive functions are the result of their ability to secrete a plethora of neuroactive products, cytokines and chemoattractants such as histamine, serotonin, neuropeptides, nerve growth factor, interferon-γ, transforming growth factor-β, interleukin-1, interleukin-6, tumor necrosis factor-α, nitric oxide, lipid metabolites (prostaglandins, leukotrienes, platelet-activating factor) and enzymes (mast-cell proteases I and II, tryptase, phospholipase, chymase)^9,10^.

Adult neurogenesis, the generation of new neurons throughout life from a pool of resident stem cells, primarily occurs in two major regions of the mammalian brain, the hippocampal dentate gyrus (DG) and the subventricular zone (SVZ) of the lateral ventricle. Nautiyal and colleagues previously reported that MC-deficient C57BL/6 c-kit^W-sh/W-sh^ mice have profound deficits in hippocampus-dependent spatial learning and memory and in baseline hippocampal neurogenesis^6^. Interestingly, the SVZ, a neurogenic niche which lacks MC under physiological conditions, was unaffected by their depletion^6^.

Concerns have been raised, however, regarding the specificity of c-kit^W-sh/W-sh^ mice as a model of MC deficiency. Activation of the c-kit receptor by its ligand stem cell factor (SCF) regulates the maturation, proliferation, survival and migration of MC whereby the c-kit^W−sh^ mutation, which affects c-kit structure, results in a constitutive lack of MC in mice^11^. Expression of c-kit however, has also been detected in many other cell types including melanoblasts, germ cells and hematopoietic stem cells^12,13^. Constitutive disruption of c-kit or SCF expression in mice thus hinders development not only of the MC lineage but also other cell types whose development is critically dependent on SCF/c-kit interactions. Likewise, several studies showed expression of SCF and c-kit in different regions of the embryonic brain and reported SCF/c-kit signaling in the stimulation of neurogenesis, neuronal survival and migration, positive chemotaxis of neurons and neurite extension during development^14–18^. In the adult mouse nervous system, SCF and c-kit expression were found in neuron-like cells in the olfactory bulb (OB), neocortex, archicortex (including hippocampus), cerebellum, brain stem and spinal cord^19^. Furthermore, c-kit is expressed by glial cells in the neocortex, hypothalamus, cerebellum and brainstem, and in nestin^+^ precursor cells in the SVZ^18^, suggesting that SCF/c-kit signaling may be involved in neuron-neuron as well as neuron-glia interactions in the mouse brain.

Given the obvious limitations of the c-kit model of MC-depletion, the aim of the present study was to use a novel, more specific MC knock-out mouse model to determine whether MC do in fact play a potential pro-proliferative or pro-neurogenic role within the two adult neurogenic niches. Here, using the MC protease (Mcpt5)CreR26^DTA/DTA^ transgenic model, which ensures very high-specificity and high-efficiency deletion of loxP-flanked DNA in connective tissue type MC, without affecting any other cell lineages^20^, we show that MC depletion in fact does not affect neural precursor proliferation or adult neurogenesis *in vivo*. Interestingly, however, we show that isolated peritoneal MC, when co-cultured at a very high density with primary adult DG or SVZ-derived cells, nevertheless can affect neural precursor proliferation and neuronal differentiation potential, an effect which is likely mediated via the release of their major chemical mediator histamine. Presumably because very few MC are found within the hippocampus (and none within the SVZ), it is unlikely that such an effect would be observed *in vivo*, at least under physiological conditions.

## Results

### MC are significantly depleted in the Mcpt5CreR26^DTA/DTA^ transgenic model

**Figure 1:**
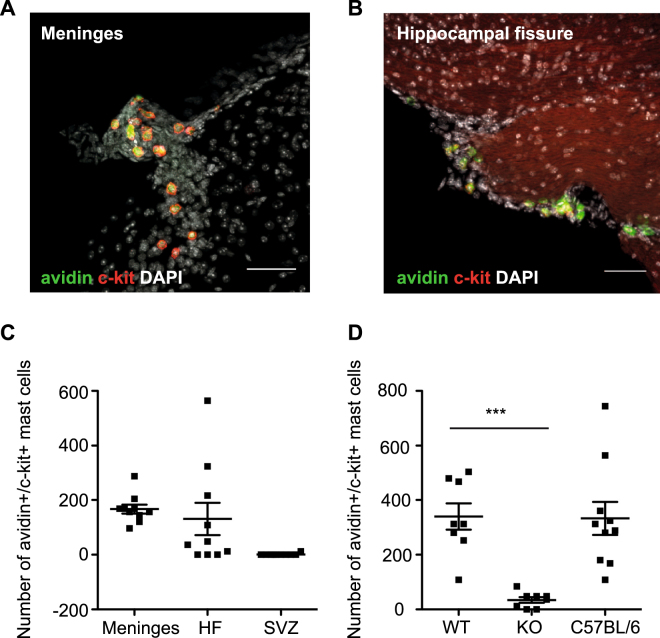
MC are present in the meninges and hippocampal fissure and are significantly depleted in the Mcpt5CreR26^DTA/DTA^ KO mice. Limited numbers of brain-resident, avidin^+^c-kit^+^ mast cells can be found in the meninges (**A**,**C**) and hippocampal fissure (**B**,**C**) of the brains of wild-type mice. (**D**) Mcpt5Cre^+^R-DTA^+^ transgenic mice have significantly fewer total brain MC than their wild-type littermates (Mcpt5Cre^−^R-DTA^+^) and C57BL/6 mice (n = 8 per group). Dunnett’s multiple comparison post-hoc test. Scale bars in A and B are 100 μm.

Given that MC have been previously shown to reside in small numbers in the brain, particularly in the regions close to the hippocampus^6^, we first aimed to quantify the MC number and localization within the brain of WT mice. We observed limited numbers of avidin^+^/c-kit^+^ MC in the meninges (166.8 ± 16.3) and hippocampal fissure (130.8 ± 59.1), but not in the SVZ (1.2 ± 1.2; Fig. 1A–C) of WT mice. The Mcpt5CreR26^DTA/DTA^ transgenic mouse model has been shown to result in dramatically fewer MC compared to WT mice in the peritoneal cavity (1.99% of WT), abdominal skin (11.1%) back skin (11.2%) and ear skin (3.5%)^20^. To confirm that the brains of Mcpt5CreR26^DTA/DTA^ knock-out mice are also depleted of MC, we analyzed MC numbers within the brain of WT and MC-KO mice. In the brains of the MC-KO mice the total number of brain-resident MC was significantly decreased to 10.1% of those found in WT littermates (WT: 339.5 ± 48.1 vs MC-KO: 34.5 ± 10.3 vs C57BL/6: 332.8 ± 60.5 MC per brain, *p* =< 0.01; Fig. 1D).

### DG and SVZ precursor proliferation are not affected by MC deficiency

**Figure 2:**
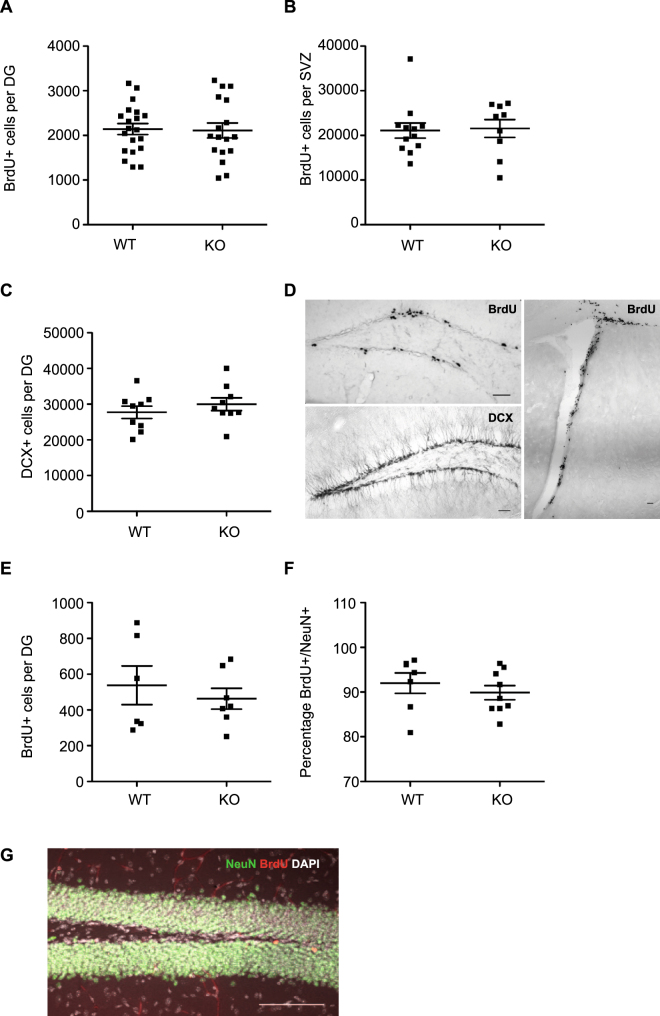
Precursor proliferation is not affected by MC deficiency. (**A**) No difference in hippocampal precursor proliferation was observed between adult Mcpt5CreR26^DTA/DTA^ knock-out (KO; n = 20) and wild-type mice (WT; n = 17). (**B**) Similarly, no difference in SVZ precursor proliferation was observed between Mcpt5CreR26^DTA/DTA^ KO (n = 12) and WT (n = 9). (**C**) No difference in the number of DCX^+^ immature neurons was observed in the DG of MC KO mice (n = 9 mice) compared to WT littermates (n = 9). (**D**) Representative images of BrdU and DCX staining in the DG and BrdU staining in the SVZ. (**E**) No difference in the survival of proliferating cells was observed in the DG of MC KO mice (n = 7 mice) compared to WT mice (n = 6 mice). (**F**) MC depletion had no effect on the percentage of surviving new-born neurons, 28 d following BrdU labeling. (**G**) Representative image of BrdU and NeuN (Fox3) staining. Scale bars, 100 μm.

To next determine whether MC are involved in regulating neural precursor proliferation under physiological conditions, adult transgenic MC-KO and WT littermates were injected with the thymidine analogue BrdU and the number of proliferating cells in the DG and SVZ were analyzed 12 h later. We found no significant difference in the number of BrdU^+^ cells in either the subgranular zone of the hippocampal DG (WT: 2142 ± 122.6 vs KO: 2109 ± 168.3, *p* = 0.87; Fig. 2A,D) or the SVZ (WT: 21086 ± 1714 vs KO: 21539 ± 1998, *p* = 0.86; Fig. 2B,D).

### DG neuronal differentiation is not affected by MC deficiency

To determine whether MC deficiency affects differentiation of proliferating precursor cells into neurons, we first quantified the number of DCX^+^ in the WT and MC-KO DG, which reflect the population of late intermediate progenitor cells and early postmitotic neurons. We found no difference in the number of DCX^+^ cells between genotypes (WT: 27708 ± 1738 vs KO: 29995 ± 1792, *p* = 0.37; Fig. 2C,D). To next evaluate the effect of MC depletion on net adult neurogenesis we labeled proliferating precursor cells in WT and MC-KO mice with a single injection of BrdU and perfused these mice after 28 days. We found no significant difference in the number of surviving BrdU^+^ cells (WT: 538 ± 108.1 vs KO: 462.9 ± 58.4, *p* = 0.54; Fig. 2E,G), or in the percentage of BrdU^+^/NeuN^+^ new-born neurons (WT: 92 ± 2.3 vs KO: 89.9 ± 1.6, *p* = 0.44; Fig. 2F,G) in the hippocampal DG.

### The c-kit^+^avidin^−^ cells in the DG are neurons

**Figure 3:**
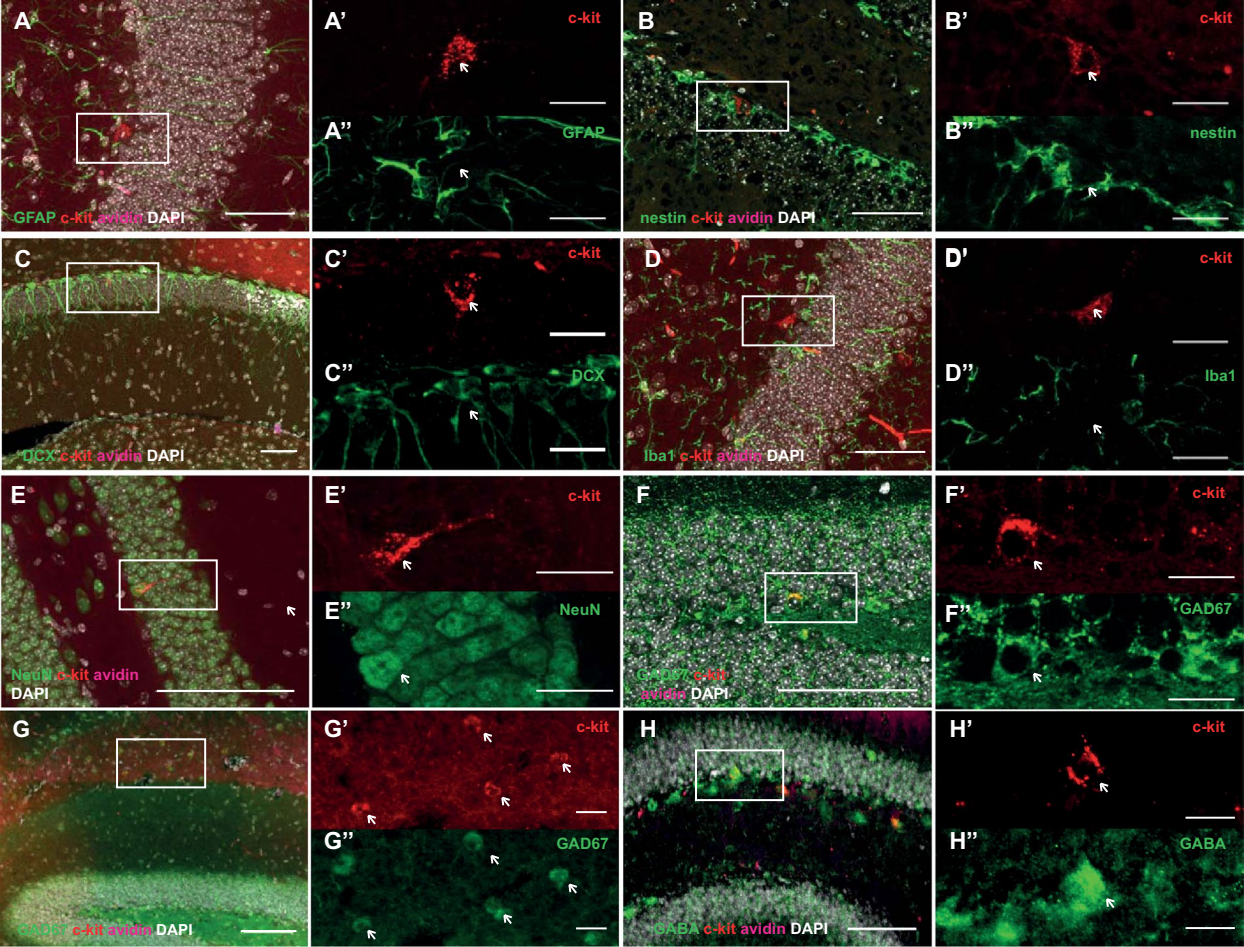
c-kit^+^avidin^−^ cells are NeuN^+^ neurons. Small numbers of c-kit^+^avidin^−^ cells can be found in the DG of adult C57BL/6 mice. These cells are not GFAP^+^ astrocytes (**A**), nestin^+^ precursor cells (**B**), DCX^+^ immature neurons (**C**), or Iba1^+^ microglia (**D**). Interestingly, the c-kit^+^avidin^−^ cells appeared to express the mature neuron marker NeuN (**E**). These c-kit^+^ neurons are GAD67^+^ (**F**), GABA^+^ (**G**) interneurons. White arrows indicate c-kit^+^avidin^−^ cells. c-kit is shown in red, avidin is magenta, DAPI^+^ nuclei are white and the cell-type-specific markers are green. Scale bars are 100 μm in main figures and 20 μm in inserts.

We identified a small population of cells positive for c-kit, but unlike MC, negative for avidin (c-kit^+^avidin^−^) in the DG of adult WT mice. While very few c-kit^+^avidin^−^ cells could be found in the cortex, olfactory bulb, and hippocampus, no c-kit^+^avidin^−^ cells at all could be identified in the SVZ. On average 190 c-kit^+^avidin^−^ cells were found per DG of all three genotypes, indicating that in our MC-deficient model the number of c-kit^+^avidin^−^ cells is not affected (C57BL/6: 189 ± 22.2 vs WT: 190.5 ± 21.6 vs MC-KO: 189 ± 38.7 c-kit^+^avidin^−^ cells per hippocampus, n = 4 mice per group). To determine the identity of these c-kit^+^avidin^−^ cells we phenotyped them with a panel of cell-type specific markers. We could show that the c-kit^+^avidin^−^ cells were not GFAP^+^ astrocytes (Fig. 3A), nestin^+^ precursor cells (Fig. 3B), DCX^+^ cells (Fig. 3C), or Iba1^+^ microglia (Fig. 3D). Interestingly, the c-kit^+^avidin^−^ cells expressed the neuronal marker NeuN (Fig. 3E). Because they were DCX-negative they could not be the immature, early postmitotic new neurons, which are DCX/NeuN double-positive. Their morphology and location also argued against their nature as granule cells. It has been previously suggested that c-kit expression in the adult mouse brain was restricted to GAD67-expressing GABAergic interneurons^21^. Further phenotying of the c-kit^+^avidin^−^ cells with a number of interneuron-specific markers indeed revealed that the majority of the c-kit^+^avidin^−^ cells in the hippocampus as well as the cortex were GAD67^+^ (Fig. 3F,G), GABAergic (Fig. 3H) interneurons.

### MC increase proliferative potential of SVZ neural precursor cells *in vitro*

We have shown that *in vivo*, under physiological conditions MC depletion does not affect neural precursor proliferation. We next wanted to determine whether MC, when co-cultured in very high numbers with neural precursor cells, are able to release factors that can influence neural stem cell proliferation and differentiation. To do this we co-cultured 2 × 10^5^ PCMCs with primary cells isolated from the two neurogenic niches using the neurosphere assay.

**Figure 4:**
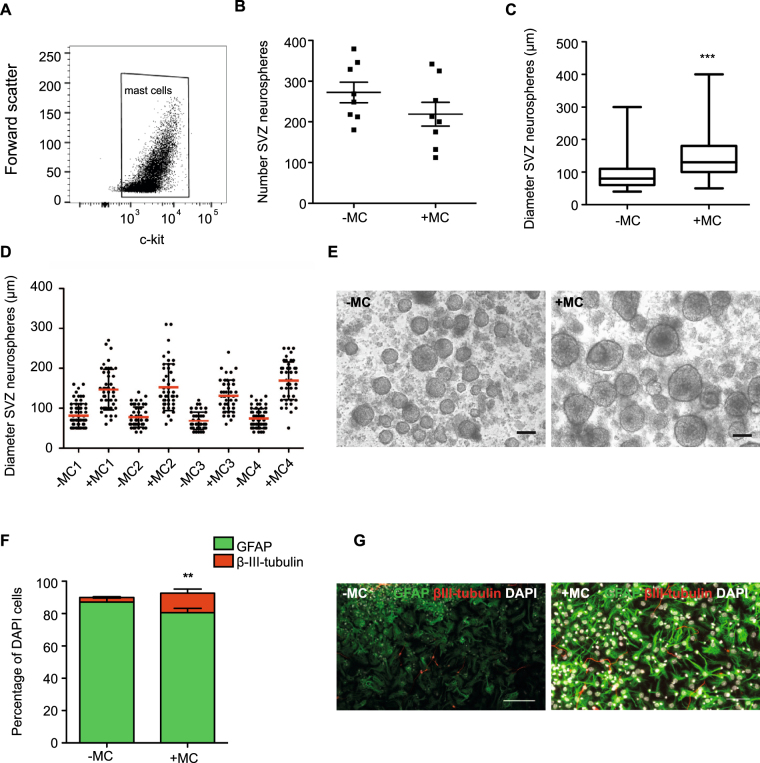
MC increase proliferative potential and neuronal differentiation of SVZ neural precursor cells. Peritoneal MC were isolated and expanded in culture. Using flow cytometry the purity of these cultures was shown to be very high (>99%, **A**). (**B**) Co-culture of MC with SVZ-derived precursor cells led to a small but not significant decrease in neurosphere number (n = 8 experiments). (**C**,**D**) The diameter of the SVZ-derived neurospheres generated in the presence of MC was significantly greater (n = 12 experiments with 50 random neurospheres sized per experiment). (**E**) Representative images of SVZ-derived neurospheres cultured in the absence or presence of MC. (**F**) Following differentiation, the SVZ-derived neurospheres cultured in the presence of MC gave rise to significantly more β-III-tubulin^+^ neurons (n = 4 coverslips per experiment with 4 random fields of view imaged per coverslip). (**G**) Representative image of SVZ neurosphere differentiation showing GFAP^+^ astrocytes in green, β-III-tubulin^+^ neurons in red and DAPI^+^ nuclei in white. Scale bars in E and G are 100 μm.

Firstly, the purity of MC in *in vitro* cultures was determined. Strong c-kit immunoreactivity is characteristic of MC as well as other cell types including neurons, astrocytes and microglia, melanoblasts, germ cells and hematopoietic stem cells, but its expression is lost in mature immune cells. Given the isolation procedure, although some limited contamination with hematopoietic stem cells is possible, hematopoietic stem cells are found at only very low numbers in the peripheral blood. The c-kit expression in cultured cells was assessed by immunofluorescence and quantified by flow cytometry. MC isolated from four C57BL/6 mice were expanded in separate cultures and analyzed. On average 99.1% of cultured cells were c-kit^+^ (Fig. 4A), indicating very high homogeneity of PCMCs. When cultured in the presence of MC (2 × 10^5^), we observed a small but not statistically significant decrease in the number of SVZ neurospheres generated (−MC: 273 ± 71; +MC: 219 ± 84, *p* = 0.059; Fig. 4B), suggesting that MC derived factors do not initiate proliferation of quiescent neural stem cells isolated from the SVZ. Interestingly, in contrast to this finding although MC are not found in the SVZ under physiological conditions, when co-cultured *in vitro* they significantly increased SVZ neurosphere diameter (Fig. 4C–E; −MC: 92.9 ± 2.2 μm vs MC + : 144.7 ± 2.6 μm, *p* < 0.0001). We also assessed neuronal differentiation in MC co-cultured SVZ neurospheres by quantifying the percentages of astrocytes (GFAP^+^) and immature neurons (β-III-tubulin^+^). Here, we observed a significant increase in the percentage of neurons in the neurospheres cultured in the presence of MC (−MC: 2.9 ± 0.46 vs +MC: 12.0 ± 2.6%, *p* = 0.0026; Fig. 4F,G).

### MC increase the number of DG-derived neurospheres

**Figure 5:**
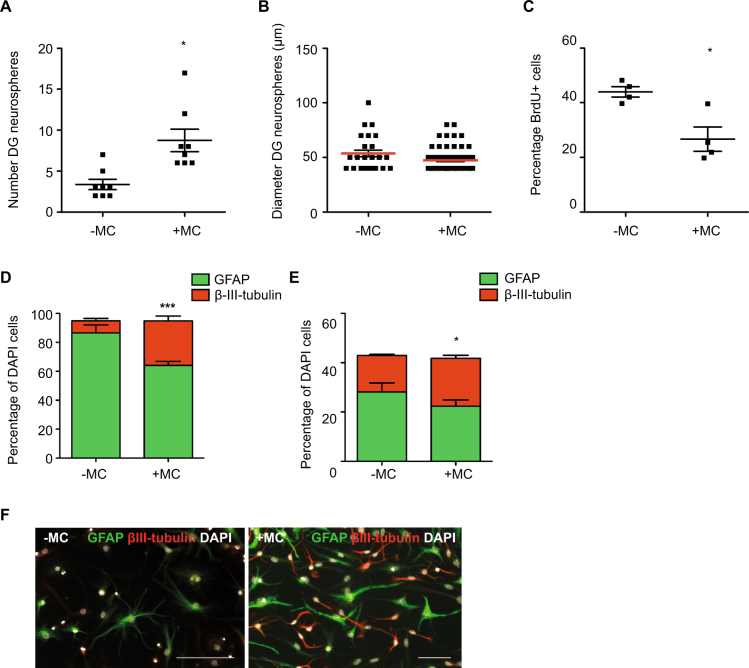
MC increase the number and neuronal differentiation potential of DG-derived neurospheres. (**A**) Co-culture of MC with DG-derived precursor cells led to a significant increase in neurosphere number (n = 8 experiments). (**B**) The diameter of the DG-derived neurospheres generated in the presence of MC however, was similar to those cultured without MC (n = 8 experiments). (**C**) The percentage of BrdU-labeled hippocampus-derived adherent cells was decreased when cultured in the presence of MC (n = 3 experiments) (**D**) Following differentiation, the DG-derived neurospheres cultured in the presence of MC gave rise to significantly more β-III-tubulin^+^ neurons (n = 5 coverslips per experiment). (**E**) Similarly, adherent monolayer cultures grown in the presence of MC generated significantly more β-III-tubulin^+^ neurons (n = 3 coverslips per experiment) (**F**) Representative image of a differentiated DG neurosphere showing GFAP^+^ astrocytes in green, β-III-tubulin^+^ neurons in red and DAPI^+^ nuclei in white. Scale bar in F is 100 μm.

When MC were co-cultured with DG derived precursor cells, we found an increase in the number of neurospheres (−MC: 3.4 ± 0.6 vs +MC: 8.8 ± 1.4, *p* = 0.01; Fig. 5A), indicating that MC-derived factors can activate normally latent DG precursor cells to divide. No effect on the diameter of DG-derived neurospheres however was observed (−MC: 53.7 ± 3.0 μm vs +MC: 47.4 ± 1.2 μm, *p* = 0.06; Fig. 5B), indicating that the proliferative capacity of the already dividing cells was not affected by MC-derived factors. Similarly, co-culture of MC with hippocampal-derived adherent monolayer cells did not increase the percentage of proliferating BrdU^+^ cells, instead leading to a small decrease in proliferation (−MC: 43.9 ± 1.9 vs +MC: 26.7 ± 4.5% BrdU^+^ cells, *p* = 0.01; Fig. 5C). Similar to the SVZ, we observed a significant increase in the potential for differentiation along the neuronal lineage of both primary neurospheres (−MC: 8.4 ± 1.7 vs +MC: 30.6 ± 3.4% β-III-tubulin^+^ cells, *p* = 0.0004; Fig. 5D,F) and adherent monolayer cells (−MC: 14.8 ± 0.5 vs +MC: 19.4 ± 1.3% β-III-tubulin^+^ cells, *p* = 0.03; Fig. 5E), indicating that MC release factors that increase neuronal-lineage differentiation.

### *In vitro* histamine treatment increases proliferation of SVZ but not DG progenitor cells

Having shown that MC-released factors can significantly increase precursor proliferation and neuronal differentiation, we next investigated whether this effect was mediated by histamine, one of the most prominent mediators released by MC. To determine the potential effect of histamine on DG and SVZ precursor proliferation and differentiation, primary SVZ and DG cells were cultured in different concentrations of histamine (1 μM and 1 mM) using the *in vitro* neurosphere assay. In addition, to determine which receptor mediates the potential histamine effect, DG and SVZ cells were cultured with the antagonists for each histamine receptor.

**Figure 6:**
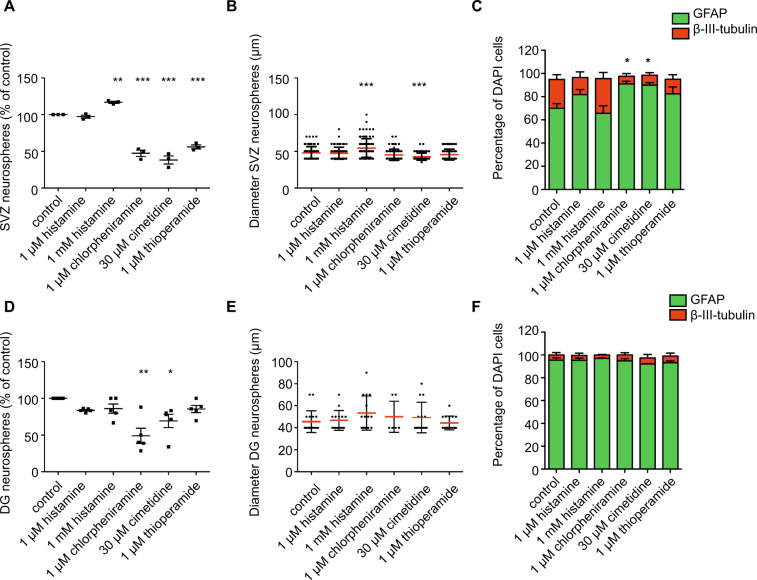
Histamine treatment increases the number, size and neuronal differentiation of SVZ-derived neurospheres. (**A**) Treatment with 1 mM histamine led to a significant increase in SVZ-derived neurosphere number, whereas all three histamine receptor antagonists caused a significant reduction in SVZ neurosphere number compared to an untreated control (n = 3 experiments; Dunnett’s multiple comparison post-hoc test). (**B**) Treatment with 1 mM histamine led to a significant increase in the diameter of SVZ-derived neurospheres, while the H2R antagonist cimetidine lead to a significant decrease in neurosphere diameter (n = 3 experiments; Dunnett’s multiple comparison post-hoc test). (**C**) Following differentiation, neurospheres cultured in the presence of either the H1R or H2R antagonists generated fewer β-III-tubulin^+^ neurons (n = 3 experiments with 4 coverslips imaged per experiment; Dunnett’s multiple comparison post-hoc test). (**D**) Treatment with either the H1R or H2R antagonists led to a significant decrease in DG-derived neurosphere number (n = 5 experiments; Dunnett’s multiple comparison post-hoc test). (**E**) None of the compounds tested affected the size (**E**) or neuronal differentiation potential (**F**) of the DG-derived neurospheres (n = 5 experiments; Dunnett’s multiple comparison post-hoc test).

Treatment with 1 mM histamine caused a significant increase in SVZ neurosphere number (116.9 ± 1.3% of control, *p* < 0.01; Fig. 6A) and diameter (control: 48.1 ± 0.8 μm vs 1 mM histamine: 54.4 ± 1.3 μm, *p* < 0.001; Fig. 6B) when compared with control cultures. Treatment with 1 μM histamine however had no effect on SVZ cell proliferation, as determined by the size of the neurospheres generated. All three histamine receptor antagonists tested, chlorpheniramine (1 μM), cimetidine (30 μM) and thioperamide (1 μM), caused a significant reduction in SVZ neurosphere number in comparison to an untreated control (chlorpheniramine: 47.4 ± 4.3%, cimetidine: 38.2 ± 5.5% and thioperamide: 56.1 ± 2.7%, *p* < 0.01; Fig. 6A). Incubation with cimetidine (30 μM) also decreased the diameter of SVZ neurospheres compared to control cultures (control: 48.1 ± 0.8 μm vs cimetidine: 42.7 ± 0.5 μm, *p* < 0.001; Fig. 6B). Following differentiation, the neurospheres cultured in the presence of 1 mM histamine showed a trend towards more neuronal lineage differentiation (control: 24.8 ± 4.2 vs 1 mM histamine: 29.7 ± 5.3% β-III-tubulin^+^ neurons), whereas the neurospheres grown in the presence of both chlorpheniramine and cimetidine generated significantly fewer neurons upon differentiation (chlorpheniramine: 6.7 ± 2.2 and cimetidine: 8.6 ± 2.2% β-III-tubulin^+^ neurons, *p* < 0.05; Fig. 6C).

At both concentrations tested, histamine resulted in a small but not significant decrease in DG neurosphere number (histamine 1 μM: 83.7 ± 1.1% of control; histamine 1 mM: 86 ± 6.4% of control, *p* > 0.05; Fig. 6D) and did not affect neurosphere size (Fig. 6E). However, treatment with chlorpheniramine (1 μM), an H1R antagonist and cimetidine (30 μM), an H2R antagonist, both resulted in a significant reduction in DG neurosphere number (chlorpheniramine: 49.1 ± 10.3%, *p* < 0.01 and cimetidine: 69.3 ± 9.0% of control, *p* < 0.05, Fig. 6D). In contrast, thioperamide (1 μM), a broad antagonist of H3R, had no effect on DG neurosphere number. None of the antagonists tested had an effect on DG neurosphere size (Fig. 6E). Neither histamine nor the antagonists had any effect on the differentiation potential of DG neurospheres (Fig. 6F).

## Discussion

In contrast to the published experiments in which baseline hippocampal neurogenesis is decreased in adult MC-deficient c-kit^W-sh/W-sh^ mice^6^, our results from the Mcpt5CreR26^DTA/DTA^ model suggest that MC deficiency does not have an effect on baseline adult hippocampal proliferation and neurogenesis *in vivo*. We cannot however conclusively rule out that the possibility that the approximately 90% reduction in MC in our model is not sufficient and that the few remaining MC (by our calculation, approximately eight per whole hippocampus) can functionally compensate for this depletion. Likewise, using the c-kit^W-sh/W-sh^ mice, although MC are fully depleted, the authors cannot definitively confirm that loss of these cells is responsible for the concomitant decrease in hippocampal neurogenesis, as altered c-kit signaling and/or suppression of c-kit expression in cell types other than MC might have confounded this finding. Nautiyal and colleagues previously reported that c-kit^W-sh/W-sh^ depletion has no effect on SVZ neurogenesis^6^. This, together with the fact that we also observed no change in SVZ precursor proliferation in the Mcpt5CreR26^DTA/DTA^ MC depletion model, makes it unlikely that MC depletion would result in a decrease in neurogenesis in this niche.

In the adult mouse nervous system, SCF and c-kit expression have been detected in multiple cell types including neurons in the OB, neocortex, archicortex, cerebellum, brain stem and spinal cord^19^, glial cells in the neocortex, hypothalamus, cerebellum and brainstem, and precursor cells in the SVZ^18^. A number of studies have shown that c-kit is expressed by the precursor cells of the adult rodent SVZ^18,22^. These c-kit-positive precursor cells were responsive to SCF signaling, increasing their proliferation following subcutaneous SCF injection^22^. Other studies have identified c-kit-positive progenitor cells in the adult olfactory mucosa, and selective depletion of these cells resulted in in decreased olfactory neurogenesis^23,24^.

High expression of c-kit in rat hippocampal CA1-CA4 regions and impaired spatial learning and memory in homozygous c-kit mutant rats has also been reported^25^. Similarly, SCF-mutant mice (*Steel*-mice) demonstrated spatial learning deficits^6,26^. These findings suggest a potential involvement of SCF/c-kit signaling in learning and memory. This was further supported by the report that a selective knockout of huntingtin-associated protein 1 (Hap1) expression led to reduced early postnatal neurogenesis in the murine hippocampus^21^. In that study, c-kit was identified as direct molecular target of Hap1, and loss of Hap1 caused downregulation of c-kit expression, resulting in decreased postnatal hippocampal neurogenesis. In our study, however, we found that microglia, astrocytes and precursor cells in the adult mouse DG were in fact not c-kit^+^.

The situation in adulthood might substantially differ from younger mice. Expression of c-kit and SCF in cultured neurons, astroglia, and microglia isolated from early postnatal mice is high^19^, whereas we found that c-kit^+^ cells in the adult brain are GFAP-negative and Iba1-negative. It has been shown that during early postnatal development (P7-P15), c-kit is expressed in hippocampal NPCs (nestin^+^/c-kit^+^), immature neurons (DCX^+^/c-kit^+^), and mature neurons (NeuN^+^c-kit^+^), whereas in 4-week-old mice c-kit expression was restricted to GAD67-expressing GABAergic interneurons even though overall expression in the hippocampus was high compared to the cortex, striatum and hypothalamus at this age^21^.

In our study, we identified a population of c-kit^+^avidin^−^NeuN^+^ cells in the dentate gyrus of 2-month-old mice, confirming that c-kit^+^ neurons remain present in the mature hippocampus. Phenotyping of these c-kit^+^ neurons with GAD67 and GABA confirmed that they are indeed a small subpopulation of c-kit^+^ GABAergic interneurons. This is in agreement with other studies that report c-kit expression in basket, stellate, and Golgi cells in the cerebellar cortex^19,27–29^ and suggests a function for c-kit in inhibitory interneurons during postnatal cerebellar development^30^. Interestingly, the c-kit–expressing neurons in the cerebral cortex and olfactory bulb are also mainly interneurons^19^. In summary, these data indicate that c-kit may have a widespread function during embryonic and early postnatal brain development that becomes more restricted during adulthood. Taken together, the above studies suggest that c-kit depletion must have wider implications, also for the hippocampus, than the depletion of MC.

We could identify MC in the meninges and the hippocampal formation of C57BL/6 mice, which is in line with previous reports of MC in the hippocampal formation of B10.PL mice^31^. To further analyze a potential interaction of MC and their released factors with neural stem/progenitor cells in the neurogenic niches, we co-cultured peritoneal MC with SVZ- and DG-derived cells *in vitro*. Our results suggest that MC-derived factors activate proliferation of quiescent DG neural stem cells. Notably, our study reveals a significant boost in proliferative potential of cycling SVZ progenitors when co-cultured with MC, indicated by a dramatic increase in neurosphere diameter. This is particularly interesting, as the presence of MC in the SVZ niche *in vivo* was never observed. These results indicate that, although MC can influence SVZ precursor proliferation *in vitro*, under physiological conditions *in vivo* this interaction is unlikely.

Since MC account for 90% of the hippocampal, and up to 50% of total brain histamine and are the main source of this neuromodulator in peripheral tissues^8,32^, we next verified the effects of histamine on SVZ- and DG-derived cells *in vitro*. Knowing that histamine actions are mediated by the activation of three different histaminergic receptors (H1R, H2R, H3R), which are widely distributed throughout the CNS^8^, we also determined which of these receptors histamine is potentially acting through in both neurogenic niches. All three histamine receptors are expressed by SVZ-derived neural stem cells^33,34^.

We observed a significant increase in SVZ-derived neurosphere number and size when treated with 1 mM histamine. This result is not consistent with previously reported effects of histamine on neonatal SVZ stem/progenitor cells where a strong pro-neurogenic effect of histamine on neonatal SVZ and embryonic cortical cell cultures was observed, without changes in proliferation^34,35^. Another report has demonstrated that chronic histamine administration in the adult SVZ *in vivo* does not induce an overall increase in cell proliferation but instead may trigger neuronal commitment of SVZ cells, and identified histamine as a crucial modulator of neuronal differentiation in the SVZ-OB axis^36^. Nevertheless, in published *in vitro* studies 500 μM was the highest histamine concentration tested, possibly indicating that elevated concentrations of histamine (1 mM) may be needed to activate SVZ cell proliferation. Furthermore, the effect of endogenous histamine was abolished when SVZ-derived cells were cultured in the presence of H1R, H2R and H3R antagonists, confirming previously published results showing that histamine actions in the SVZ may be mediated by the activation of all three histaminergic receptors. Importantly, several studies have identified histamine as a potent pro-neurogenic mediator, responsible for priming of NSC in the SVZ toward the neuronal phenotype^34–37^. This is in line with the results from our study, which showed a trend towards increased neuronal differentiation in the SVZ cells treated with 1 mM histamine and a significant decrease in those treated with the H1R and H2R antagonists.

We found all three histamine receptors to be expressed in the DG (our unpublished results). In addition, a recent study demonstrated expression of the H3R in the hippocampus and showed that S38093, a novel histamine H3R antagonist promoted hippocampal neurogenesis in 3-month-old mice and improved context discrimination in aged mice^38^. In accordance with this study, we found a small but non-significant reduction in DG neurosphere number following histamine treatment. Similar to the SVZ however, we observed a significant decrease in DG-derived neurosphere number when cells were cultured in the presence of the H1R and H2R antagonists. Surprisingly however, we found no effect of the H3R antagonist.

Despite a lack of *in vitro* data for the hippocampus, previous *in vivo* experiments revealed impaired spatial learning and reduced adult hippocampal neurogenesis in H1R-knockout mice^39^. Both the aforementioned study and our own identify a potential role of endogenous histamine in adult hippocampal neurogenesis that might be executed by H1R activation. However, the exact mechanisms responsible for the reduced number of newborn cells in the hippocampus of H1R-KO mice, as well as in DG- derived cell cultures after chlorpheniramine treatment, have not been yet been sufficiently explored.

It is worth to note that the neurosphere assays described above were performed in the absence of exogenous histamine indicating that endogenous histamine may be present in our cultures. It is possible that mast cells, or other histamine releasing cells such as basophils and lymphocytes^40,41^, may be present at small numbers in our cultures. Non-mast cell derived histamine may be found in several tissues, including the brain, where it functions as a neurotransmitter^42^. In addition, MC granular contents have also been shown to diffuse a great distance, and their granules may be seen up to 500 μm away from the cell of origin^43,44^, potentially explaining why histamine may be present in regions of the brain that are devoid of MC (i.e. the SVZ).

In summary, we are just beginning to understand the role that immune cells play in regulating adult neurogenesis under physiological conditions. Here, we show using the Mcpt5CreR26^DTA/DTA^ MC-deficient mouse line that MC are not required for the maintenance of normal baseline adult neurogenesis, even though the main factor they release potentially influences adult neurogenesis. Based on our results, together with previously published data, we hypothesize that the observed decrease in hippocampal neurogenesis and deficits in hippocampus-dependent spatial learning and memory in c-kit^W-sh/W-sh^ mice^6^ may be, at least partly, the result of disrupted SCF/c-kit signaling in the neurogenic niche and may not be directly connected with MC-deficiency. While both MC and their chemical mediator histamine have the potential to influence adult neural stem cell proliferation and differentiation when supplied at supraphysiological conditions *ex vivo*, in the steady-state adult brain MC are unlikely to affect neural stem cell proliferation and adult neurogenesis.

## Materials and Methods

### Animals and housing

Mcpt5Cre^−^R26^DTA/DTA^ and Mcpt5Cre^+^R26^DTA/DTA^ mice were bred to obtain MC knock-out (MC-KO) and wild-type (WT) littermates for neurogenesis studies^20^. Mice were housed in standard cages, with 2 or 3 animals per cage and maintained on a 12 h light/dark cycle with food and water provided *ad libitum*. All experiments were conducted in accordance with the applicable European and national regulations (Tierschutzgesetz) and approved by the responsible authority (Landesdirektion Sachsen).

### BrdU injection and tissue preparation

At 8 weeks of age, MC-KO mice and WT mice received a single intraperitoneal injection of 50 mg/kg 5-bromo-2-deoxyuridine (BrdU). To measure precursor proliferation, mice were sacrificed 12 h later and to measure neuronal survival mice were sacrificed after 28 d. Mice were deeply anesthetized and perfused transcardially with 0.9% NaCl, followed by 4% PBS buffered paraformaldehyde (PFA). Afterwards, brains were fixed for 24 h in 4% PFA at 4 °C, washed in PBS and incubated in 30% sucrose dissolved in PBS. Coronal 40 μm thick sections were cut throughout the entire brain on a microtome (Leica).

### Staining, quantification and image analysis

For a list of all antibodies used in this study see Table S1. For diaminobenzadine (DAB) detection, sections were first incubated in 0.6% hydrogen peroxide diluted in PBS for 30 min at room temperature (RT) then in 2.5 M HCl for 30 min at 37 °C (BrdU staining only). After rinsing in PBS, sections were blocked in PBS++ (0.2% triton-X100 and 10% donkey serum (DS)) for 60 min at RT. Sections were incubated overnight (ON) at 4 °C in primary antibodies diluted in PBS+ (0.2% triton-X100 and 3% DS), washed extensively with PBS and incubated for 3 h at RT in secondary antibodies diluted in PBS+ . For the fluorescently-labeled sections, a 10 min incubation with 4′,6-diaminidono-2-phenylindole (DAPI; 1:1000 in PBS) was included prior to the final PBS wash. After rinsing in PBS, fluorescently-labeled sections were mounted with Aqua-Poly/Mount (Polysciences Inc). The DAB-labelled sections were incubated for 1 h at RT in Vectastain ABC-Elite (Vector Laboratories), washed, incubated for 5 min at RT with DAB (Vector Laboratories), cleared with Neo-Clear (Merck) and mounted in Neo-Mount (Merck). DAB-labelled cells were counted in the subgranular zone (SGZ) of the hippocampus and in the SVZ using an upright light microscope (DM750; Leica). Fluorescence images were acquired on the Zeiss Apotome Axio Imager Z1 microscope using either the Plan-APOCHROMAT 20x/0,75 or Plan-APOCHROMAT 40x/0,95 objectives and AxioVision (V 4.8.2.0) software. For cell phenotyping optical Z-sectioning was performed at 1 μm intervals. Images were analysed on merged z-stacks in single optical sections and maximum z-projected images were created using Fiji software (http://fiji.sc). All microscopic quantification was performed blinded to the experimental conditions.

### Isolation and culture of peritoneal-cell derived mast cells (PCMCs)

Mice were anaesthetized small incision was made below the abdomen of the mouse and 5 ml of ice cold sterile PBS was injected into the peritoneum. The peritoneal liquid containing PCMCs was collected and centrifuged. The pellet was re-suspended in RPMI medium containing 10% fetal calf serum, 50 units/ml penicillin/streptomycin, 2 mM L-Ala L-glutamine (Merck), 1 mM sodium pyruvate (Merck) and 50 μM 2-mercapto-ethanol supplemented with interleukin-3 (10 ng/ml) and stem cell factor (30 ng/ml). Cells were transferred into a T25 culture flask and cultured at 37 °C, 5% CO_2_. At day 2 the non-adherent cells were removed and 5 ml of fresh, supplemented RPMI medium was added to the culture flask. At day 6 an additional 5 ml of supplemented RPMI medium was added. At day 10, non-adherent PCMCs were obtained from culture medium for further experiments.

### Assessment of cultured MC purity by flow cytometry analysis

Cultured PCMCs were fixed and permeabilized with 4% PFA, 0.1% triton X-100 in PBS for 15 min at RT. Subsequently, cells were washed and incubated for 30 min at RT in rat anti-c-kit diluted in 1% bovine serum albumin and 0.1% triton X-100 in PBS. Afterwards, cells were washed, incubated with secondary donkey anti-rat Cy3 and re-suspended in PBS. Unstained and secondary only controls were also prepared. Samples were analyzed by flow cytometry (FACS AriaIII; BD Biosciences) using FACSDiva Software. Dead cells and cell debris were gated out on the basis of their forward and side scatter profile. c-kit^+^ cells were designated as MC.

### MC and neural precursor cell co-culture

The SVZ and the DG were dissected from brains of 8-week-old C57BL/6 mice as described previously^45^. Isolated DG and SVZ neural precursor cells were resuspended in neurosphere growth medium (neural basal medium supplemented with 1x B27, 1x GlutaMAX, 2 µg/ml heparin, 50 units/ml penicillin/streptomycin) supplemented with 20 ng/ml purified mouse receptor-grade epidermal growth factor (ProSpec), and 20 ng/ml recombinant bovine fibroblast growth factor (ProSepc)) and seeded into a 24 well plate (500 μl/well). Hippocampal adherent monolayer cultures were initiated as described previously^45^ and used between passages 7–9. Transwell inserts (Sigma-Aldrich) were placed in every second well and 2 × 10^5^ PCMCs were seeded per transwell. After 2 days the transwells were removed. At day 7 (SVZ) and day 14 (DG) neurospheres were quantified, sized and transferred to a 24-well plate containing the neurosphere growth medium without growth factors and a poly-D-lysine/laminin coated coverslip. To label proliferating adherent precursor cells, a pulse of BrdU (10 μg/ml) was added to the wells and BrdU^+^ cells stained as described above. Neurospheres were differentiated for 7 d and adherent cultures for 5 d at 37 °C with 5% CO_2_, in growth medium devoid of growth factors, then fixed with 4% PFA for 15 min at 37 °C.

### Histamine neurosphere assays

Isolated DG and SVZ neural precursor cells from C57BL/6 mice were resuspended in neurosphere growth medium and seeded into 96 well plates (200 μl/well). Cells were treated with 1 μM histamine, 1 mM histamine, 1 μM chlorpheniramine (H1R antagonist), 30 μM cimetidine (H2R antagonist), 1 μM thioperamide (H3R antagonist) or an untreated control. 48 wells were used per condition. Plates were incubated at 37 °C with 5% CO_2_. At day 7 (SVZ) and day 14 (DG) neurospheres were counted, sized and differentiated.

### Statistical Analysis

Experimental results were analysed using a Student’s t-test unless otherwise stated. Statistical significance was depicted as (*)*p* < 0.05, (**)*p* < 0.01 and (***)*p* < 0.001.

### Data availability

All data generated and analysed during this study are included in this published article

## Electronic supplementary material

Table S1
