# Author Correction: Mast cells increase adult neural precursor proliferation and differentiation but this potential is not realized *in vivo* under physiological conditions

**DOI:** 10.1038/s41598-020-76589-y

**Published:** 2020-11-09

**Authors:** Joanna M. Wasielewska, Lisa Grönnert, Nicole Rund, Lukas Donix, Ruslan Rust, Alexander M. Sykes, Anja Hoppe, Axel Roers, Gerd Kempermann, Tara L. Walker

**Affiliations:** 1 CRTD – Center for Regenerative Therapies Dresden, Technische Universität Dresden, Dresden, Germany; 2 German Center for Neurodegenerative Diseases (DZNE) Dresden, Dresden, Germany; 3 Brain Research Institute ETH and University of Zurich, Zurich, Switzerland; 4 Max Planck Institute for Molecular Cell Biology and Genetics, Dresden, Germany; 5 Institute for Immunology, Medical Faculty Carl Gustav Carus, Technische Universität Dresden, Dresden, Germany

Correction to: *Scientific Reports* 10.1038/s41598-017-18184-2, published online 19 December 2017

The original version of this Article contained errors in the spelling of the authors Joanna M. Wasielewska, Lisa Grönnert, Nicole Rund, Lukas Donix, Ruslan Rust, Alexander M. Sykes, Anja Hoppe, Axel Roers, Gerd Kempermann and Tara L. Walker which were incorrectly given as J. M. Wasielewska, L. Grönnert, N. Rund, L. Donix, R. Rust, A. M. Sykes, A. Hoppe, A. Roers, G. Kempermann and T. L. Walker.

These errors have now been corrected in the PDF and HTML versions of the Article.

