# Supplementary material for: Mast cells increase adult neural precursor proliferation and differentiation but this potential is not realized in vivo under physiological conditions
Source: Sci Rep. 2017 Dec 19;7:17859. doi: 10.1038/s41598-017-18184-2 (PMC5736663; doi:10.1038/s41598-017-18184-2)
Supplement: Supplementary file 1 — Table S1 [file 41598_2017_18184_MOESM1_ESM.doc]

**Supplementary data**

**Mast cells increase adult neural precursor proliferation and differentiation but this potential is not realized *in vivo* under physiological conditions**

Wasielewska JM^1,2^, Grönnert L^1,2^, Rund N^1,2^, Donix L^1^, Rust R^3^, Sykes, AM^4^, Hoppe A^5^, Roers A^5^, Kempermann G^1,2^ and Walker TL*^1,2^

^1^ CRTD – Center for Regenerative Therapies Dresden, Technische Universität Dresden, Dresden, Germany

^2^ German Center for Neurodegenerative Diseases (DZNE) Dresden, Dresden, Germany

^3^ Brain Research Institute ETH and University of Zurich, Zurich, Switzerland

^4^ Max Planck Institute for Molecular Cell Biology and Genetics, Dresden, Germany

^5^ Institute for Immunology, Medical Faculty Carl Gustav Carus, Technische Universität Dresden, Dresden, Germany

**Supplementary Table 1. List of Antibodies**

| Primary antibodies | Species | Dilution | Company |
| --- | --- | --- | --- |
| βIII-tubulin | Rabbit | 1:2000 | Covance |
| BrdU | rat | 1:500 | Serotec |
| c-kit | rat | 1:500 | ThermoFisher Scientific |
| DCX | goat | 1:250 | Santa Cruz |
| Fox3 (NeuN) | rabbit | 1:1000 | Abcam |
| GFAP | Mouse | 1:500 | Dako Cytomation |
| GFP | rabbit | 1:500 | ThermoFisher Scientific |
| Iba1 | rabbit | 1:1000 | Wako Chemicals |
| NeuN | Mouse | 1:1000 | Millipore |
| Directly-coupled antibodies |  |  |  |
| FITC-avidin |  | 1:100 | Biolegend |
| Secondary antibodies |  |  |  |
| anti-mouse Cy3 | donkey | 1:500 | Jackson ImmunoResearch |
| anti-goat AlexaFluor647 | donkey | 1:500 | Jackson ImmunoResearch |
| anti-goat biotin | donkey | 1:500 | Jackson ImmunoResearch |
| anti-rabbit AlexaFluor 488 | donkey | 1:500 | Jackson ImmunoResearch |
| anti-rabbit AlexaFluor 647 | donkey | 1:500 | Jackson ImmunoResearch |
| anti-rat biotin | donkey | 1:500 | Jackson ImmunoResearch |
| anti-rat Cy3 | donkey | 1:500 | Jackson ImmunoResearch |
